# A Snake Venom Peptide and Its Derivatives Prevent Aβ_42_ Aggregation and Eliminate Toxic Aβ_42_ Aggregates *In Vitro*

**DOI:** 10.1021/acschemneuro.4c00089

**Published:** 2024-07-03

**Authors:** Luana Cristina Camargo, Ian Gering, Mohammadamin Mastalipour, Victoria Kraemer-Schulien, Tuyen Bujnicki, Dieter Willbold, Mônika A. Coronado, Raphael J. Eberle

**Affiliations:** † Institute of Biological Information Processing (IBI-7: Structural Biochemistry), Forschungszentrum Jülich, Jülich 52428, Germany; ‡ Faculty of Mathematics and Natural Sciences, Institute of Physical Biology, Heinrich Heine University Düsseldorf, Düsseldorf 40225, Germany

**Keywords:** peptide, snake venom, Aβ_42_, aggregation, deaggregation, d-peptide

## Abstract

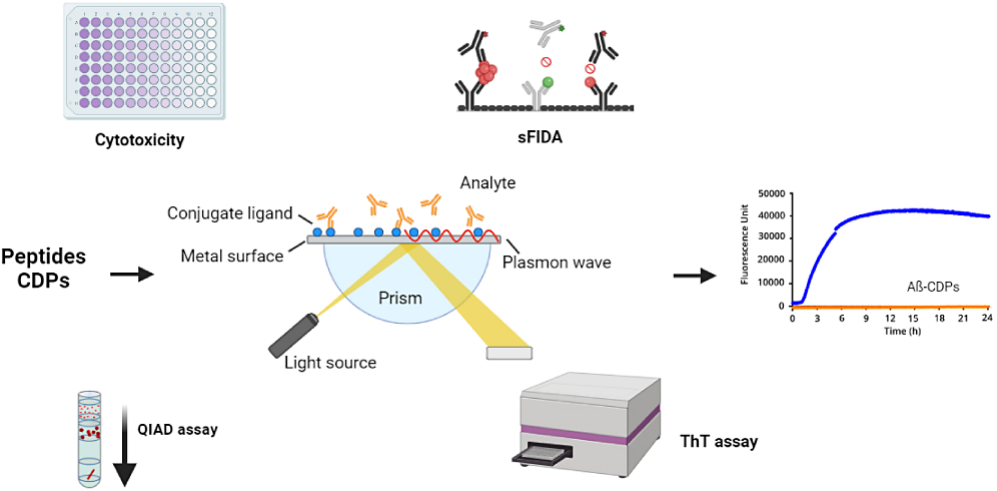

Over a century has passed since Alois Alzheimer first described Alzheimer’s disease (AD), and since then, researchers have made significant strides in understanding its pathology. One key feature of AD is the presence of amyloid-β (Aβ) peptides, which form amyloid plaques, and therefore, it is a primary target for treatment studies. Naturally occurring peptides have garnered attention for their potential pharmacological benefits, particularly in the central nervous system. In this study, nine peptide derivatives of Crotamine, a polypeptide from *Crotalus durissus terrificus* Rattlesnake venom, as well as one d-enantiomer, were evaluated for their ability to modulate Aβ_42_ aggregation through various assays such as ThT, QIAD, SPR, and sFIDA. All tested peptides were able to decrease Aβ_42_ aggregation and eliminate Aβ_42_ aggregates. Additionally, all of the peptides showed an affinity for Aβ_42_. This study is the first to describe the potential of crotamine derivative peptides against Aβ_42_ aggregation and to identify a promising d-peptide that could be used as an effective pharmacological tool against AD in the future.

## Introduction

The increase in life expectancy today can be associated with a higher incidence of age-related diseases, such as Alzheimer’s disease (AD).^1−3^ AD is known to affect elderly by inducing cognitive deficits as well as operational impairment.^4^ Consequently, AD patients initially experience difficulty with daily tasks, which progressively leads to a complete dependence on caretakers. Currently, only one drug, called lecanemab, is approved by the Food and Drug Administration (FDA) as a curative treatment of AD.^5^

The hallmarks of AD are the presence of neuritic plaques, neurofibrillary tangles, and neurodegeneration.^6−9^ The first above-mentioned structures are composed of protein aggregates, which then induce the observed neurodegeneration. Neuritic plaques are primarily composed of amyloid-β (Aβ) misfolded peptides. These peptides assemble into oligomers, described as the most toxic conformation, and eventually form into fibrils.^10−14^ Aβ is produced by the sequential cleavage of Amyloid Precursor Protein (APP) by different secretases.^15^ First, APP is cleaved at the C-terminal part of the protein by β-secretase, then Aβ is formed from APP cleavage by γ-secretase.^16−18^ This cleavage produces different Aβ isoforms, among which Aβ (1–42) (Aβ_42_) is one of the most toxic and prone to aggregation.^19,20^

Considering that Aβ aggregation seems to be the initial downstream event in AD and the fact that only one drug, lecanemab, has been approved that directly interacts with Aβ, the development of new drugs targeting this protein remains essential. Since the beginning of civilization, natural compounds have played a significant role in treating various diseases, including neurological disorders. Therefore, the naturally occurring peptides may hold an important place in the drug development against AD.^21^ In this context, snake venom has been studied for its potential compounds with antimicrobial and anticancer properties. Beyond these properties, some snake venom compounds have already been approved for treating high blood pressure (Captopril) and as antiplatelet (Tirofiban and Eptifibatide).^22^ In the central nervous system, snake venom compounds are known to interact with distinct receptors, reducing pain, neuroinflammation, anxiety, and depression.^23^

Crotamine, which is a protein isolated from *Crotalus durissus terrificus*, has different positive biological effects; when injected in the hippocampus, crotamine has been shown to improve cognition in rats.^24^ Additionally, this polypeptide possesses cell-penetrating properties, which can play a role in drug delivery.^25,26^ For years, many cell-penetrating peptides have been studied for AD treatment due to their nontoxic and high activity properties. Crotamine has two specific regions that enable it to translocate quickly and efficiently into actively proliferating cells.^26^ These regions are classified as nucleolar targeting peptides (NrTPs). Based on the literature, we selected the amino acids regions from Lys27 to Lys39,^26,27^ which retain some properties of crotamine and it is smaller in size.

In this study, we evaluated peptides derived from crotamine (Lys27–Lys39). Subsequently, some of those amino acids were replaced to improve the peptide performance. CDPs and one of its d-enantiomer were investigated for their ability to (1) prevent Aβ_42_ aggregation; (2) eliminate Aβ_42_, and (3) exhibit affinity to Aβ_42._ The results we are describing here suggested that CDPs could serve as potential lead peptides targeting Aβ_42_ aggregation.

In the context of advances in biotechnology, d-enantiomeric peptides present a solution to the challenges associated with peptides in clinical applications. They are resistant to proteases and exhibit lower immunogenicity.^28^ Several distinct d-peptides have been investigated for Alzheimer’s disease treatment, showing promising results.

## Results and Discussion

### Naturally Occurring Peptides Prevent Aβ_42_ Aggregation

**Figure 1:**
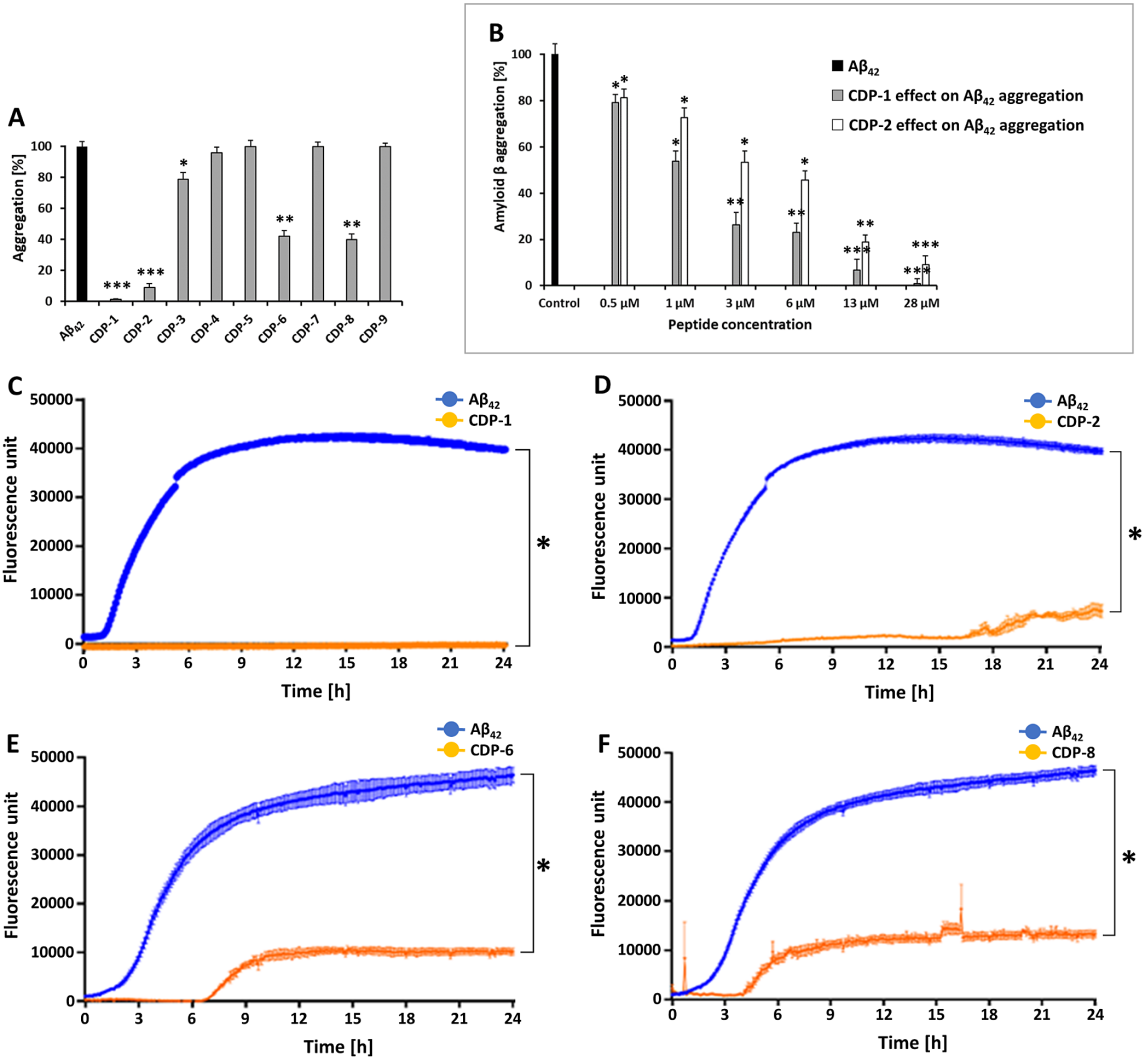
The effect of CDPs on Aβ_42_ aggregation using Thioflavin T assays. The ThT fluorescence signal with only Aβ_42_ is shown in blue. In orange is the action of CDP-1, -2, -6, and -8 in the signal of Thioflavin T, which decreased over time. (A) Provides an overview of the effect of the tested peptides against Aβ_42_ aggregation. (B) Demonstrates the effect of different doses of CDP-1 and CDP-2 (0.5, 1, 3, 6, 13, and 28 μM) on Aβ_42_ aggregation. The end point is shown for the relative fluorescence during a ThT assay. The Aβ_42_ aggregation serves as the control. (C) The effect of CDP-1 against Aβ_42_ aggregation. (D) The effect of CDP-2 against Aβ_42_ aggregation. (E) The effect of CDP-6 against Aβ_42_ aggregation. (F) The effect of CDP-8 against Aβ_42_ aggregation. Data shown are the mean ± SEM from three independent measurements (*n* = 3). Asterisks mean that the data differ from the Aβ_42_ control significantly at *p < 0.05, **p < 0.01, and ***p < 0.001 levels according to analyses by two-way ANOVA.

Thioflavin T-based assays are used for the *in vivo* and *in vitro* detection of amyloid aggregates. First, the potential of the eight CD peptides (CDPs) to reduce Aβ_42_ aggregation was evaluated at a concentration of 28 μM. Four CDPs showed a significant decrease of the ThT signal compared to Aβ_42_ alone (Figures 1A and S3). The strongest effect could be observed for CDP-1, where no Aβ_42_ aggregation was determined over the experimental time (Figure 1C; two-way ANOVA, *F* (1440, 4800) = 5,349; *p* < 0.0001). CDP-2, -6, and -8, were able to reduce the aggregation, however, not in the same proportion indicated in the negative control. The signal was four times smaller than in the control (Figure 1D–F; two-way ANOVA, *F* (1440, 4800) = 5,349; *p* < 0.0001). The CDP-1 peptide had the strongest reduction potential over the incubation time, where no Aβ_42_ aggregation was detected (Figure 1C).

Additional ThT experiments were performed to evaluate the dose dependency of the CDP-1 and CDP-2 peptides, as they revealed the most substantial effect against the Aβ_42_ aggregation. The results of those experiments demonstrated the dose response relationship of the peptides. The graph in Figure 1B effectively communicates the percentage reduction in the Aβ_42_ aggregation after 24 h, showing that CDP-1 was much more effective compared to CDP-2 (Figures 1B and S4).

### Effect of CDPs on Aβ_42_ Oligomer Size Distribution Using QIAD

**Figure 2:**
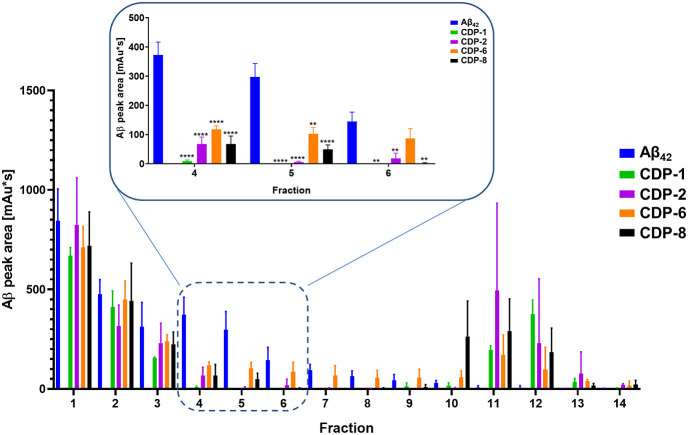
CDP derivatives eliminated the Aβ_42_ oligomers. In the QIAD assay, the Aβ_42_ solution was separated into different fractions according to the particle size. All peptides were able to reduce the toxic Aß oligomers. When Aβ_42_ was incubated with CDP-1, CDP-2, CDP-6, and CDP-8, there was a reduction in the peak area corresponding to Aβ_42_ in the HPLC chromatogram. The data presented the mean ± SEM obtained from three independent measurements (*n* = 3). Asterisks denote significant differences from the control group at varying levels of significance. Specifically, * represents *p* < 0.05, ** represents *p* < 0.01, *** represents *p* < 0.001, and **** represents *p* < 0.0001, as determined by analyses conducted through two-way ANOVA.

ThT analysis indicated that four crotamine-derived peptides, CDPs (CDP-1, -2, -6, and -8), eliminated or efficiently decreased Aβ_42_ aggregation. To quantify the effect of the CDPs on the Aβ_42_ oligomer and aggregate size distribution, we performed QIAD assays. For this assay, RP-HPLC was performed to disassemble all of the different Aβ_42_ assemblies. The oligomer elimination efficiency is defined as the reduction of Aβ_42_ contents in fractions 4 to 6 in the presence of the CDPs. Fractions 4–6 containing the Aβ_42_ oligomers were explicitly sensitive to the studied peptides (Figure 2). The CDPs proved to be significantly efficient.

As observed in the ThT assay, Aβ_42_ was not detected in fractions 4–5 when incubated with CDP-1, CDP-2, CDP-6, and CDP-8 (two-way ANOVA, *p* < 0.001; *F* (12, 39) = 2,276) compared to the control (Figure 2). In fraction 6, however, Aβ_42_ was detectable even after incubation with CDP-6 (two-way ANOVA; *p* = 0.7985).

### Surface-Based Fluorescence Intensity Distribution Analysis Assay to Follow Aβ_42_ Oligomer Elimination

**Figure 3:**
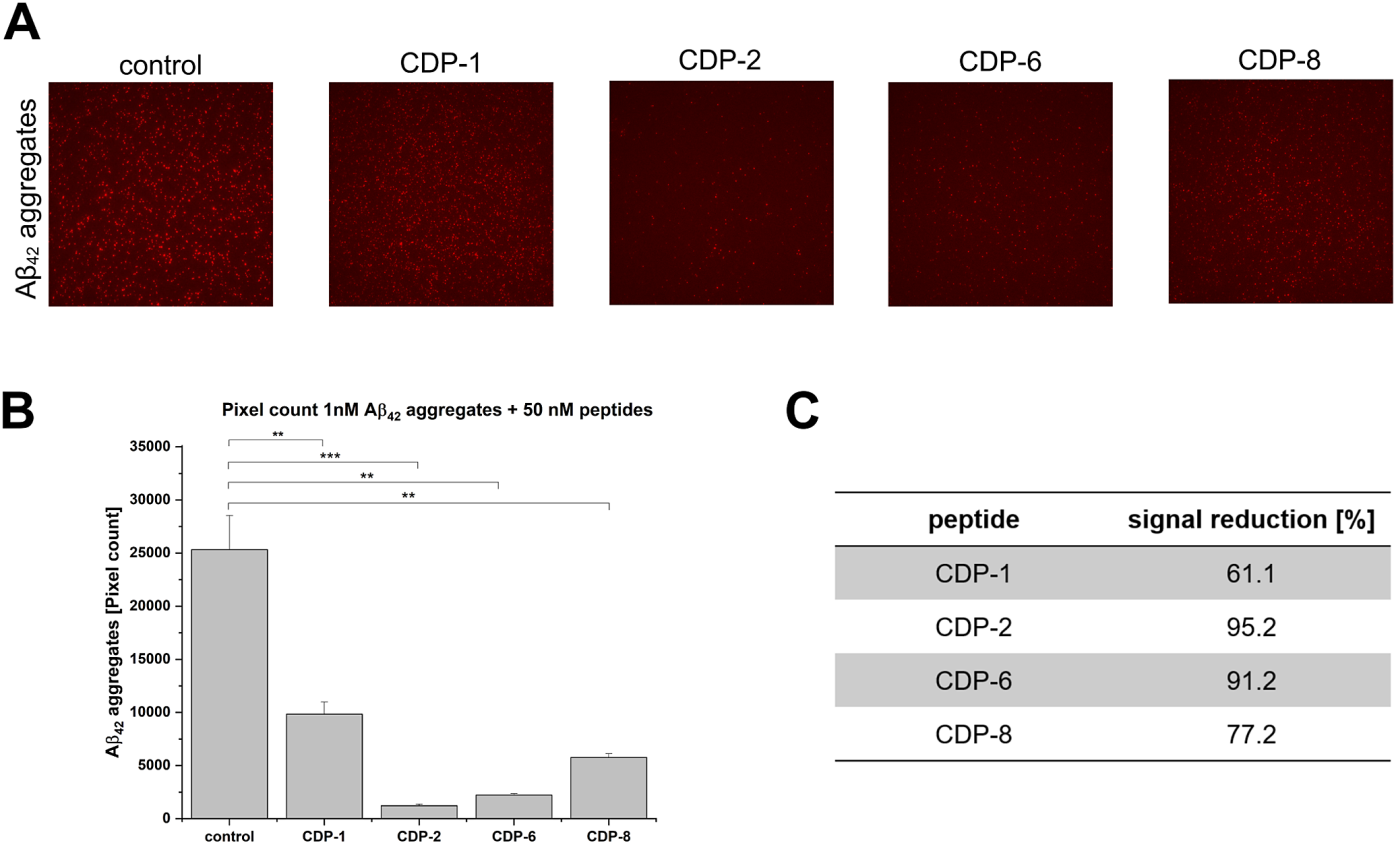
sFIDA experiments to follow Aβ_42_ aggregates elimination by CDPs. Antibody Nab228 was captured on the plate surface. After incubation of the samples, the Aβ targets were detected with IC-16 labeled with CF633. The assay surface was then imaged with the TIRFM. (A) TIRFM images of 1 nM Aβ_42_ aggregates (1 nM) were treated with 50 nM CDPs or without CDPs (control). (B) Pixel count analysis of the TIRFM images from A. One nM Aβ_42_ aggregates treated with 50 nM CDPs or without CDPs (control). (C) Each peptide’s signal reduction compared with the control (1 nM Aβ_42_ aggregates). The data presented represent the mean ± SEM from three independent measurements (*n* = 3). Asterisks denote significant differences from the control group, where ** indicates *p* < 0.01 and *** indicates *p* < 0.001 levels of significance, as determined by analyses conducted through a two-sample *t* test.

Surface-based fluorescence intensity distribution (sFIDA) employs a biochemical setup similar to that of ELISA-like techniques. However, sFIDA uses the same epitope for capturing and detecting antibodies, leading to only recognizing oligomers and aggregates without detecting monomers.^29^ The microscopy-based readout ensures single-particle sensitivity.^29^ sFIDA was performed to demonstrate the ability of selected CDPs to eliminate Aβ_42_ aggregates through a different methodology. Initially, analysis of Aβ_42_ aggregates at different concentrations was carried out (Figure S5). To follow the elimination of Aβ_42_ aggregates by the peptides, 50 nM of CDP-1, CDP-2, CDP-6, and CDP-8 were incubated, separately, with 1 nM Aβ_42_ aggregates (Figure 3). It could be observed that all samples containing the studied peptides have a reduction of the Aβ aggregates, with the most substantial effect for CDP-2 with a reduction of 95.2%, followed by CDP-6 with a 91.2% reduction in the aggregate (Figure 3A–C).

sFIDA experiments demonstrated that CDP-1, -1D, -2, -6, and -8 eliminate Aβ_42_ aggregates. However, the effect of CDP-1 and its d-enantiomer was not pronounced as observed in the ThT and QIAD assay experiments. Further adjustment and optimization of the sFIDA assay conditions are in progress.

### Determination of CDP-Binding Affinities with Aβ_42_

The interaction kinetics of CDP-1, -2, -6, and -8 with Aβ_42_ was determined using surface plasmon resonance (SPR) experiments. Equilibrium dissociation constant (*K*_D_) of the peptides CDP-1, -2, -6, and -8 was determined under the assay described in Material and Methods. Aβ_42_ was immobilized via covalent primary amino group coupling, and CDP-2, -6, or -8 peptides were injected as analytes. In the case of CDP-1, the peptide was immobilized, and Aβ_42_ were the analyte. Figure S6 shows the SPR sensorgrams and saturation curves for the tested peptides. The affinity interaction was determined using steady-state model. All peptides were able to interact with Aβ_42_, although with varying affinity (Figure S6 and Table 1). CDP-1 reveled the lowest *K*_D_ and therefore, the highest affinity for Aβ_42_ with a *K*_D_ value of 406.8 nM; our findings unveiled a better affinity compared to a widely studied peptide, D3D3, a head-to-tail tandem version of D3, a fully d-enantiomeric peptide targeting Aβ_42_ (N, O), followed by CDP-2, which exhibited a *K*_D_ value of 3.25 μM, falling within a comparable range of affinities observed with D3 and RD2 (derived from D3) d-enantiomer peptides. Interestingly, CDP-6 (*K*_D_ 26.38 μM) and CDP-8 (*K*_D_ 569.6 μM) exhibited a very lower affinity to Aβ_42_ than CDP-1, suggesting that the Cys residue plays a crucial role in shaping the secondary structure of the peptides.

**Table 1:** *K*_D_ Values Were Determined by SPR Experiments

steady-state fitting	
peptide	***K*_D_ ± STD (μM)**
CDP-1	0.41 ±
CDP-2	3.25 ± 0.2
CDP-6	26.28 ± 5.7
CDP-8	569.6 ±

SPR results revealed substantial differences between CDP-1 and its derivatives CDP-2, -6, and -8, which may be related to the change in the secondary structure of the derivatives compared to the original CDP-1 peptide. CD experiments conducted on the peptides demonstrated differences in the secondary structure of CDP-1 compared to CDP-2, -6, and -8. These differences are likely to impact the binding behavior of the peptides with Aβ_42_ (Figure S9). The secondary structure analysis based on the CD results, using the BeStSel online tool,^30,31^ revealed a notable decline in α helix content among the peptide variants. For instance, while CDP-1 exhibited a significant α helix content of 25%, this characteristic diminished in subsequent derivatives such as CDP-2, -6, and -8 (<7%) (Table S1). The loss of structural composition suggests a nuance alteration in the peptide’s conformational landscape, potentially influencing its interaction dynamics, as already described for CDPs targeting the SARS-CoV-2 protease.^27^ Further, Jiang and collaborators (2019) described α-helical peptide inhibitors against Aβ oligomer formation. Their findings underscore the correlation between the loss of secondary structure and the functionality of these inhibitors.^32^

### Effect of CDP-1 d-Enantiomer against Aβ_42_ Aggregation and Toxic Aβ_42_ Aggregates

**Figure 4:**
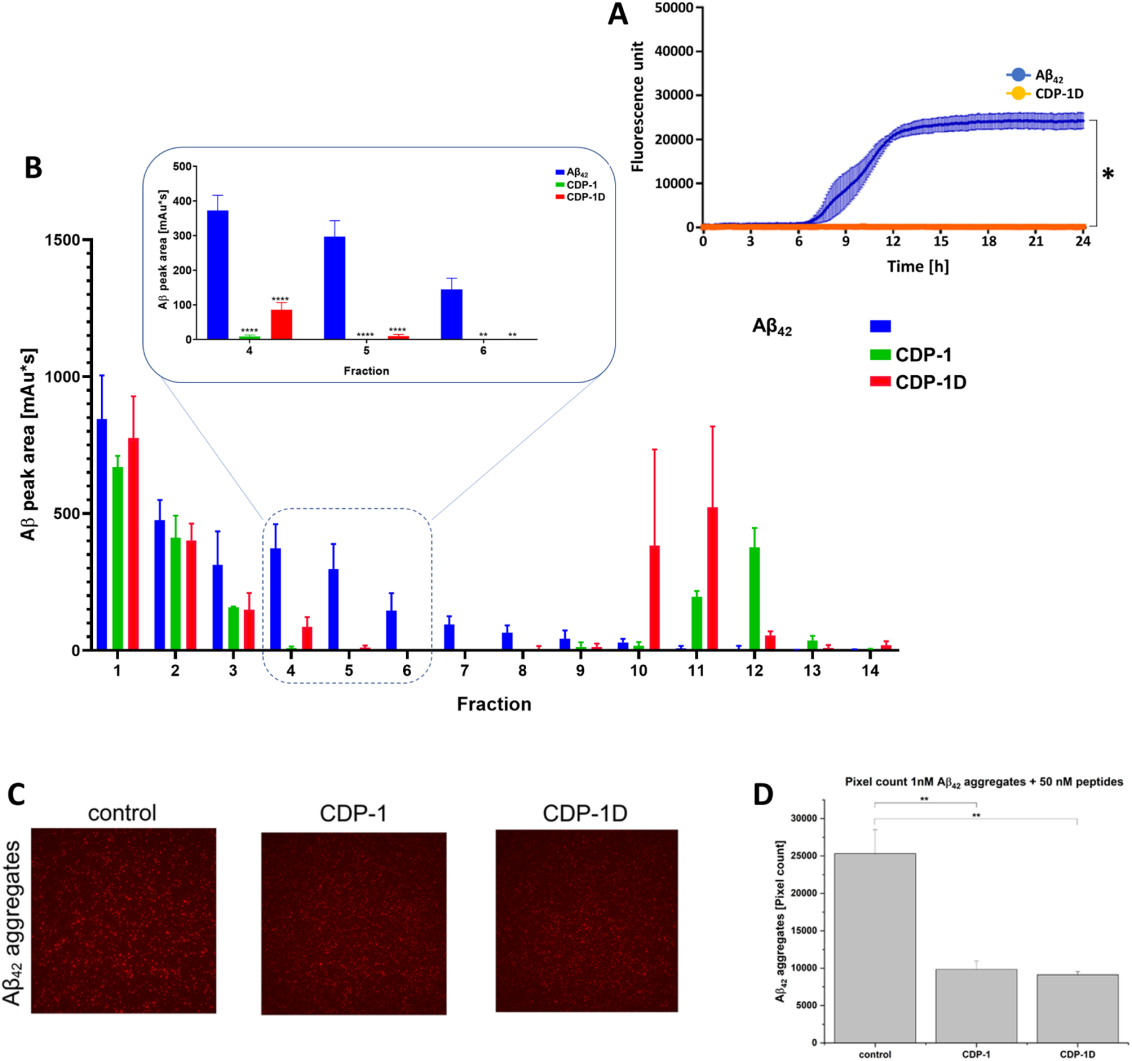
The effect of CDP-1D against Aß_42_ aggregation and toxic Aß_42_ aggregates. (A) D-CDP-1 inhibits Aβ_42_ aggregation in the Thioflavin T assay (orange). The ThT fluorescence signal with only Aβ_42_ increased over time (blue). (B) Eliminated Aβ_42_ oligomers in the QIAD assay. The results are shown in comparison with the mother l-peptide CDP-1. (C) sFIDA assay results for CDP-1 and CDP-1D. TIRFM images of Aβ_42_ aggregates treated with CDP-1 and CDP-1D. (D) Pixel count analysis of the TIRFM images from C. The data presented represent the mean ± SEM from three independent measurements (*n* = 3). Asterisks denote significant differences from the control group, where ** indicates *p* < 0.01 levels of significance, as determined by analyses conducted through a two-sample *t* test.

Peptides are attractive drug candidates and have increasingly become the leading molecules in drug development. However, their application is limited due to the susceptibility of l-peptides to endogenous enzymes. On the contrary, peptides composed of d-amino acids are rarely accessible to these enzymes. d-Peptides, when compared with their l-enantiomeric counterparts, possess several therapeutic advantages. As shown previously, the proteolytic stability of d-peptides is superior to l-peptides, which can significantly extend the serum half-life,^33,34^ resulting in reduced immunogenicity and increased bioavailability of the d-peptides.^35^ CDP-1 exhibited the most promising results in inhibiting Aβ_42_ aggregation. Therefore, building upon the aforementioned findings, CDP-1 was synthesized in its d-enantiomeric form, designated as CDP-1D, and subsequently assessed for its potential in mitigating Aβ_42_ aggregation and eliminating toxic aggregates using ThT, QIAD, and sFIDA assays (Figure 4).

Like CDP-1, the d-enantiomeric form of the peptide showed the potential to inhibit Aβ_42_ aggregation by 100% at the tested concentration of 28 μM in the ThT assay (Figure 4A). To demonstrate the efficacy of the d-enantiomeric peptide, we also applied QIAD assay’s. We demonstrate that both peptides showed efficiency in eliminating the Aβ_42_ aggregates. However, contrary to the results reported for CDP-1, Aβ_42_ aggregates were detected after CDP-1D treatment (Figure 4B), and we suspect that CDP-1D agent yielded significant reduction of Aβ_42_ oligomers in fraction 6; however, it was not able to eliminate it by 100%. The sFIDA experiment revealed a decrease in aggregates after CDP-1D treatment, detected in the same quantity described for the l-enantiomeric counterparts (Figure 4C).

PRI-002, D3, RD2D3, and their cyclic forms demonstrated the ability to reduce Aβ aggregation in the ThT assay and eliminate Aβ oligomers in the QIAD assay.^36−38^ In a similar vein, CDP-1D demonstrated the capacity to reduce Aβ_42_ aggregation by 0% and to eliminate Aβ_42_ oligomers. Therefore, the chiral modification did not affect the efficacy of CDP-1D *in vitro*, suggesting its potential as a reliable candidate for *in vivo* treatment studies. Currently, the majority of treatment studies prioritize the disruption of Aβ_42_ aggregation due to its significant role in Alzheimer’s disease pathology, toxicity in AD.^19^ Aβ_42_ is known to be a toxic species and, therefore, was chosen in this study. In the preclinical stages of AD, where clinical symptom are absent, Aβ_42_ is already present in the brain, transitioning from monomeric form to oligomers, and ultimately fibrils.^11,13,14^ The different stages can be evaluated in both QIAD and ThT assay.^39,40^

### Cytotoxicity Assay of CDPs against SH-SY5Y and HEK293 Cells

**Figure 5:**
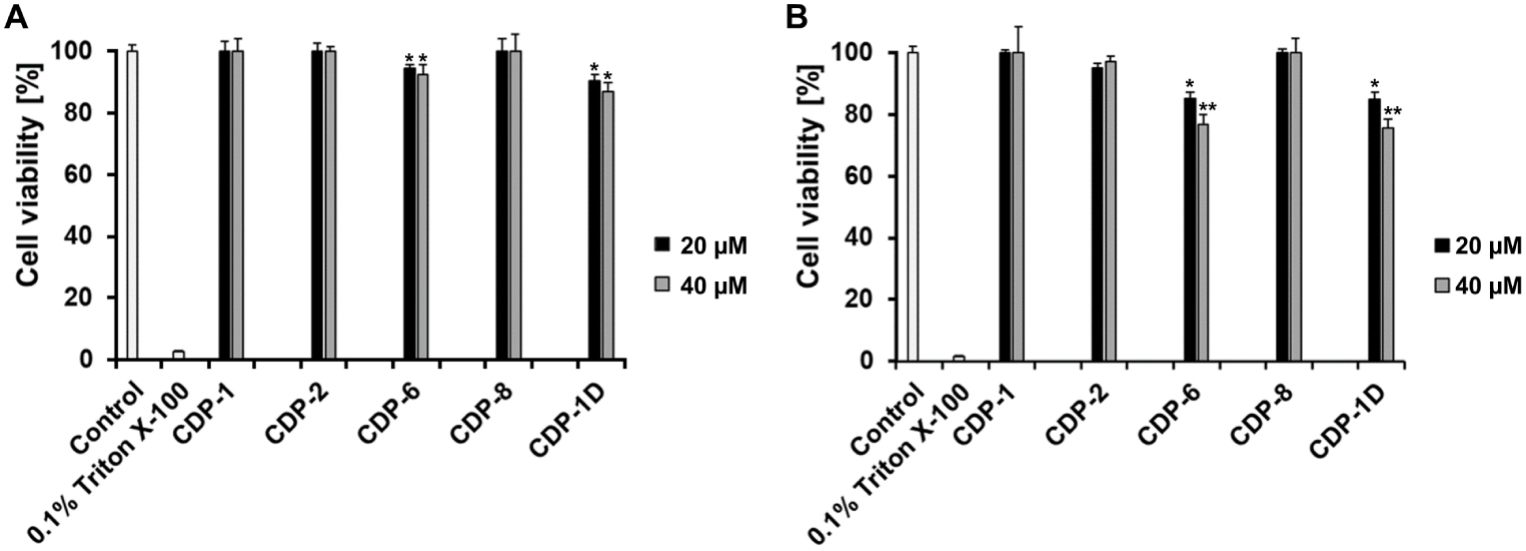
MTT assay of CDPs in SH-SY5Y and HEK293 cells. MTT assay evaluated the cytotoxicity of four l-peptides and one d-peptide. The effects of 20 and 40 μM peptides on the viability of both cell lines are shown. The complete MTT assay (concentrations tested between 0 and 100 μM) for each peptide is shown in Figures S7 and S8. The control shows the cell viability without peptide, and 0.1% Triton X-100 was used as the negative control. (A) SH-SY5Y cell line and (B) HEK293 cell line. The data displayed represent the mean ± SEM from three independent measurements (*n* = 3). Asterisks indicate significant differences from the control group, where * represents *p* < 0.05 and ** represents *p* < 0.01 levels of significance, as determined by analyses conducted through two-way ANOVA.

Different concentrations of CDP-1, -2, -6, -8, and -1D were evaluated regarding a cytotoxic effect (Figures S7 and S8). A cytotoxicity assay was performed aiming the safety of the peptides using two different cells: SH-SY5Y (Human neuroblastoma) and HEK293 (Human embryonic kidney) cells. Figure 5A,B displays the viability of SH-SY5Y and HEK293 cells treated with 20 and 40 μM of each peptide, respectively. The peptide concentration determination represents the same and 1.5- to 2-fold concentrations used in the ThT (28 μM) and QIAD (20 μM) assays.

The MTT assay revealed that CDP-1, CDP-2, and CDP-8 were nontoxic to both tested human cell lines at 20 and 40 μM concentrations (Figure 5); even at high test dose (100 μM), the SH-SY5Y cell viability was higher than 90% for the tested peptides: CDP-1: 98%; CDP-2: 94.4%, and CDP-8: 91.4% (Figure S7). In comparison, at the same peptide concentration, the viability of HEK293 cells was significantly reduced to 66% (CDP-1), 53% (CDP-2), and 64% (CDP-8), which demonstrated dose-dependent toxicity, with significant reductions in HEK293 cell viability at 100 μM (Figure S8). The cell viability of CDP-6 and CDP-1D (20 and 40 μM final concentrations) tested in SH-SY5Y cells was >85% and for HEK293 cells >70%. At higher concentration (100 μM), the cell viability of SH-SY5Y was reduced, >75% (CDP-6: 86% and CDP-1D: 78%). The peptides’ toxicity was assessed at an elevated concentration (100 μM) in HEK293 cells, resulting in an anticipated substantial decrease in cell viability: CDP-6 exhibited 48% viability, while CDP-1D showed 47%. Our results clearly illustrate that the chosen peptides exhibit different effects depending on both the concentration and cell type, which can be explained due to differences between cell types, tissue origin, and biological function. The literature extensively discusses how the choice of tissue or cell type used in a study can alter the performance and results of cytotoxicity and/or cell viability assays.^41^ Cell viability and cytotoxicity assays rely on diverse cellular functions, such as cell membrane permeability, enzyme activity, cell adherence, ATP production, coenzyme production, and nucleotide uptake activity.^42^ The latter may contribute to the heightened cytotoxicity of CDP-1D compared to its l-enantiomeric counterparts (CDP-1) at high doses. d-Peptides exhibit lower enzyme sensitivity relative to l-peptides and may lead to more pronounced adverse effects *in vitro*.^43−45^

In this study, we report a novel capability to mitigate Aβ_42_ aggregation and facilitate the dissolution of Aβ_42_ aggregates *in vitro.* Crotamine, a polypeptide isolated from *Crotalus durissus terrificus*, has been the subject of extensive study for many years. Crotamine can cross membranes and have anticancer properties.^26^ Besides, crotamine improved memory in rats when infused intrahippocampally.^24^ The crotamine derivative peptides (CDPs) used in this study also demonstrated inhibitory potential against both SARS-CoV-2 cell culture and the virus’s main protease *in vitro*.^27^ CDP-1 was initially conceived as a segment of the crotamine polypeptide, whereas CDP-2 and CDP-8 were derivatives with cysteine/serine substitution. Additionally, based on the original CDP-1 peptide, a d-enantiomer peptide named CDP-1D was synthesized. d-Enantiomeric peptides are considered useful tool in the drug development since those peptides are resistant to proteases degradation and less immunogenic.^46,47^ Several publications have previously demonstrated the potential of peptides to eliminate Aβ aggregation and, to a lesser extent, to disassemble Aβ aggregates (Table 2). Among these peptides are synthetically developed d-enantiomers.

**Table 2:** Peptide Inhibitors Targeting Aβ

**peptides**^a^	**prevent Aβ_42_ aggregation**	**eliminate Aβ_42_ aggregates**	**references**
RYYAAFFARR	yes	-	(48)
pgklvya and kklvffarrrra	yes	-	(49)
FDYKAEFMPWDT	yes	-	(50)
Ac-LPFFN-NH2	yes	-	(51)
KLVFF and variants	yes	-	(52,53)
MLRTKDLIWTLFFLGTAVS	yes	-	(54)
KFFEAAAKKFFE and variants	yes	-	(55)
TLWYK, EHWYH, HYFKY, HYYIK, and KYYEI	yes	-	(56)
AFRADVRAERAE and variants	yes	-	(32)
γ-AApeptides	yes	yes	(57)
lLwHsK and sHwHsK	yes	yes	(58)
rprtrlhthrnr, rprtrlhthrnrrprtrlhthrnr, ptlhthnrrrrrrprtrlhthrnr, and ptlhthnrrrrr	yes	yes	(36,39,59−61)
CDPs	yes	yes	Described in this manuscript

a Capital letters corresponds to l-amino acids and small letters to d-amino acids in the peptide sequences.

Further experiments are required to unravel the precise mechanism by which CDPs inhibit Aβ aggregation and facilitate the elimination of Aβ aggregates. However, depending on the charge distribution on the surface of the molecules, we suggest that the cationic CDPs (with a net charge of +5) could potentially interact with negatively charged regions present on the surface of the Aβ monomer, oligomer, or fibrils. The N-terminal region of the Aβ monomer (Asp1-Lys16) contains four negatively charged residues (Asp1, Glu3, Asp7, and Glu11). Along with Glu23 and Asp23, they form a negatively charged surface that might be attractable to interact with the positively charged CDP residues (Figure S10). Several studies demonstrated a particular contribution of the Aβ N-terminus to its aggregation behavior.^62^ Building on this knowledge, we hypothesize that CDPs may interact with the Aβ N-terminus, thereby impeding the aggregation process. This interaction potentially disrupts key steps in the aggregation pathway, representing a promising avenue for therapeutic intervention against Alzheimer’s disease.

Further studies have revealed that in large Aβ aggregates and mature fibrils, the N-terminus becomes exposed while C-terminus remains concealed.^63,64^ The N-terminal domain plays a crucial role in shaping the structures of aggregates and fibrils. Residues of the N-terminus form essential salt bridges during fibril assembly, such as the interaction between Asp1-Lys28 and Asp7 with Arg5.^64^ These salt bridges contribute significantly to the stability and architecture of the fibrils. Besides, the N-terminal portion of Aβ(1–10) forms a β-sheet structure by binding with Aβ (12–22) within fibrils.^65^ The interaction of CDPs with the N-terminus not only serves to prevent aggregation but also has the potential to destabilize oligomers or aggregates, leading to their elimination. This effect was explored by Mallesh et al. 2023, who investigated how peptides interact with Aβ_42_ and their antiaggregation effects, which were characterized by a reduction in β-sheet formation.^52^

CDP-1 exhibited an nM affinity for interacting with Aβ_42_, efficiently eliminated toxic Aβ_42_ oligomers in the QIAD assay, and completely inhibited ThT-positive Aβ_42_ fibrils *in de novo* ThT aggregation assays. Based on this performance, CDP-1D, the d-enantiomer of CDP-1, displayed promising effects in preventing Aβ_42_ aggregation and eliminating toxic Aβ_42_ aggregates. Additionally, MTT assays revealed either no or minimal cytotoxicity of the peptides against SH-SY5Y and HEK293 cells at concentrations used in the ThT and QIAD assays (ranging from 20 to 28 μM). However, at a concentration exceeding 60 μM, the CDPs exhibited significant cytotoxic effects against HEK293 cells compared to SH-SY5Y cells, as demonstrated in this study. Similar cytotoxic effects were observed in Vero cells^27^ and NIH-3T3 cells.^66^ It is anticipated that the sensitivity of different cell lines to cytotoxic effects of the same compound will vary.^67−69^ Despite this, the properties of CDP-1 and CDP-1D make them promising lead peptides, as they advance to the next stage of the development process.

## Material and Methods

### Peptides

Synthetic crotamine derivative peptides (CDP) were synthesized by Genscript (Leiden, NL) with a purity of >95%. HPLC chromatograms demonstrate the purity of each peptide (Figures S1 and S2). The peptides were acetylated at the N-terminus and methylated at the C-terminus. Essential information about the CDPs used in this study is summarized in Table 3. All peptides were diluted in water in a stock solution of 500 mM and placed at 4 °C until further use. Aβ_42_ (Bachem, Bubendorf, Switzerland) was suspended in HFIP (1 mg/mL) overnight, lyophilized, and stored at room temperature.

**Table 3:** Basic Information about the Tested Peptides

**name**	**sequence**	**conformation**	**solvent**
CDP-1	KMD**C**RWRWK**CC**KK	L	H_2_O
CDP-2	KMD**S**RWRWK**SS**KK	L	H_2_O
CDP-3	KMD**C**RWRWK**SS**KK	L	H_2_O
CDP-4	KMD**S**RWRWK**CC**KK	L	H_2_O
CDP-5	KMD**S**RWRWKS**C**KK	L	H_2_O
CDP-6	KMD**S**RWRWK**C**SKK	L	H_2_O
CDP-7	KMD**C**RWRWKS**C**KK	L	H_2_O
CDP-8	KMD**C**RWRWK**C**SKK	L	H_2_O
CDP-1D	kk**cc**kwrwr**c**dmk	D	H_2_O

### Circular Dichroism Spectroscopy of CDPs

Circular dichroism measurements were carried out with a Jasco J-1100 Spectropolarimeter (Jasco, Germany). Far-UV spectra were measured at 190 to 260 nm using a peptide concentration of 30 μM in ddH_2_O. The secondary structures of CDPs (1 to 8) and CDP-1D were checked. A 1-mm path length cell was used for the measurements; 10 repeat scans were obtained for each sample, and five scans were conducted to establish the respective baselines. The average baseline spectrum was subtracted from the average sample spectrum. The results are presented as molar ellipticity [θ], according to eq 1:
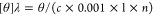
1where θ is the ellipticity measured at the wavelength λ (deg), *c* is the peptide concentration (mol/L), 0.001 is the cell path length (cm), and *n* is the number of amino acids. The secondary structure determination was performed using the BeStSel online tool (A,B).

### Thioflavin T Assay

To evaluate the ability of the peptides to prevent Aβ_42_ aggregation, a ThT assay was performed. Aβ_42_ (10 μM), ThT (5 μM), and the peptides (28 μM) were incubated in 96-well plates for 24 h at room temperature in a plate reader (Clariostar, BMG Labtech, Ortenberg, Germany). During this time, the ThT fluorescence was measured every 6 minutes with an excitation of 440 nm and emission of 490 nm. The data were corrected considering the blank wells (without Aβ_42_ and the peptides). None of the peptides interact with ThT alone or has autofluorescence properties. All measurements were performed in triplicate (*n* = 3), and data are presented as mean ± SM.

### Investigation of CDP Doses Dependency on the Aβ_42_ Aggregation Process

Different concentrations were titrated to investigate CDP-1 and -2 dose dependency on the Aβ_42_ aggregate formation, and a ThT assay was performed as described before. The effect of 0, 0.5, 1, 3, 6, 13, and 28 μM peptides was tested against 10 μM Aβ_42_ over 24 h. All experiments were performed in triplicate (*n* = 3), and data are presented as mean ± SM.

### Quantitative Determination of Interference with Aβ_42_ Aggregate Size Distribution (QIAD)

In order to evaluate the efficacy of the peptides in eliminating Aβ_42_ oligomers, QIAD assays were performed.^39^ Briefly, lyophilized Aβ_42_ (80 μM) was incubated for 2 h in sodium phosphate buffer for Aβ_42_ oligomerization. Then, each peptide (20 μM) was added to the Aβ_42_ solution and incubated for 30 min. Finally, samples were added on top of an iodixanol density gradient (5–50% (*w*/*v*) (OptiPrep, Sigma-Aldrich, Darmstadt, Germany) and centrifuged for 3 h at 4 °C and 259,000 x g (Optima TL-100, Beckman Coulter, Brea, CA, USA). For the sample analysis, 14 fractions (140 μL each) were collected from top to bottom. The top fractions, named fractions 1–2, contained Aβ_42_-monomers; fractions 4–6 contained the Aβ_42_-oligomers, which are of special interest; and the bottom fractions 11–14 contained high molecular weight of aggregated Aβ_42_. Each density gradient fraction was analyzed by Reversed Phase Liquid Chromatography. For this an Agilent 1260 Infinity II (Santa Clara, California, USA) system was equipped with an Agilent Zorbax SB-300 C-8 5 μm, 4.6 × 250 mm Column (Santa Clara, California, USA) and a multi-wavelength detector set to acquire the UV absorbance at 214 nm. H_2_O + 0.1% trifluoroacetic acid (AppliChem, Darmstadt, Germany) and acetonitrile (Roth, Karlsruhe, Germany) + 0.1% trifluoroacetic acid were used as eluent A and B, respectively. The acquisition method consisted of an initial isocratic step at 15% B, followed by a gradient from 15% B to 45% B in 10 min and another isocratic step at 45% B. The column temperature was set to 80 °C for the entire analysis. This method ensured full separation of the iodixanol density gradient medium and Aβ_42_ in all fractions. Aβ_42_ peaks were analyzed and integrated by the Agilent OpenLab 2.5 software. All measurements were performed in triplicate (*n* = 3), and data are presented as mean ± SM.

### Surface Plasmon Resonance

The dissociation constant (*K*_D_) of CDP-2, CDP-6, and CDP-8 binding to Aβ_42_ was determined by SPR spectroscopy using a Biacore T200 instrument (Cytiva, formerly GE Healthcare, Uppsala, Sweden). Aβ_42_ was immobilized on a series S CM-5 sensor chip (Cytiva, Uppsala, Sweden) by amino coupling. For this, two flow cells of the chip were activated with a freshly prepared solution containing 50 mM *N*-hydroxysuccinimide (NHS) and 16.1 mM *N*-ethyl-*N*′-(dimethylaminopropyl)carbodiimid (EDC) (XanTec, Düsseldorf, Germany) for 7 min. Aβ_42_ which was stored as a 1 mg/mL solution in HFIP was lyophilized and resolved in 10 mM sodium acetate pH 5.0 buffer (Merck, Darmstadt, Germany) to a final concentration of 50 μg/mL. The Aβ_42_ solution was injected over one of the two activated flow cells until a signal of 900 RU was reached. After immobilization of Aβ_42_, both flow cells were deactivated by a 7 min injection of 1 M ethanol at pH 8.5 (XanTec, Düsseldorf, Germany). The activated and deactivated flow cells without Aβ_42_ served as a reference.

Multicycle kinetic experiments were performed with 10 mM HEPES + 50 mM NaCl + 0.05% Tween 20 (AppliChem, Darmstadt, Germany) as the running buffer at 25 °C at a flow rate of 30 μL/min flow rate. The peptides were diluted in running buffer to final concentrations ranging from 50 to 0.02 μM with 1:2 dilution steps. Each sample was injected for 360 s, followed by a dissociation time of 600 s with running buffer. After each sample, the chip was regenerated with a 45 s injection of 2 M guanidinium hydrochloride (AppliChem, Darmstadt, Germany). The chip was allowed to equilibrate with a running buffer before the next sample injection. The reference flow cell and buffer injections (*c* = 0 nM) were used to double reference the sensorgrams. Data were evaluated and fitted to a steady-state affinity model with the Biacore T200 Evaluation Software 3.2.

The experimental setup was slightly changed to determine the *K*_D_ for binding of CDP-1 with Aβ_42_. Here, CDP-1 was immobilized by the procedure as mentioned above to a signal of 1916.5 RU. For multicycle experiments, Aβ_42_ was used as the analyte in concentrations ranging from 1.85 μM to 0.02 μM with 1:2 dilution steps with running buffer. All buffers and injection times were the same for the measurement of CDP-2, CDP-6, and CDP-8, except that the dissociation time was extended from 600 to 900 s. The data were fitted to a 1:1 kinetic fit implemented in the Biacore T200 Evaluation Software, with a global Rmax value.

### Surface-Based Fluorescence Intensity Distribution Analysis Assay

To determine the influence of peptides on Aβ_42_ aggregates, an sFIDA assay was performed. The principle was previously described.^29^ Therefore, we used pretreated 384 glass bottom microtiterplates (Sensoplate plus, Greiner Bio-One GmbH, Frickenhausen, Germany) to immobilize the capture-antibody Nab228 (Sigma-Aldrich, St. Louis, Missouri, USA) in a concentration of 2.5 μg/mL in 0.1 M carbonate buffer (Carl Roth, Karlsruhe, Germany). After an overnight incubation at 4 °C, the plate was washed five times with TBS and blocked with 80 μL of a 0.5% BSA solution in TBS with 0.03% ProClin 300 (Sigma-Aldrich, St. Louis, Mo, USA) for 1.5 h at room temperature.

The influence of 50 nM CDPs on 1 nM Aβ_42_ aggregates (prepared according to Ref (70)) in PBS was determined by incubating them overnight at room temperature and 600 rpm. As a control, the same Aβ_42_ aggregates concentration was incubated without additions of peptides; instead, the buffer was added to contain the same end concentrations. The plate was washed as previously described, and 20 μL each of the peptide solution and a serial dilution of Aβ_42_ aggregates standards in PBS were added and incubated for 2 h at room temperature.

Afterward, 20 μL of 0.625 μg/mL IC-16 CF633 (Heinrich Heine Universität Düsseldorf, Germany) in TBS with 0.1% BSA was added to each well and incubated for 1 h at room temperature. This red fluorescently labeled detection antibody was labeled with CF633 succinimidyl ester (Sigma-Aldrich, St. Louis, Missouri, USA) after the manufacturer’s protocol. After a washing step to remove the redundant antibodies, 80 μL of TBS containing 0.03% Proclin were added.

By using a total internal reflection microscopy (TIRFM) (Leica Camera AG, Wetzlar, Germany), measurement (excitation: 635 nm, emission filter: 705/22 nm) was carried out with an oil immersion objective in 100× magnifications as previously described by Kass et al. 2022, and for each well, 25 of 14-bit grayscale images were measured with a size of 1000 × 1000 pixel each. These data were analyzed with our developed sFIDAta software tool.^29^ All measurements were performed in triplicate (*n* = 3), and data are presented as mean ± SM.

### Cell Viability Assay

The cell viability assay was performed using the reduction of [3-(4,5-dimethylthiazol-2-yl)-2,5-diphenyl tetrazolium bromide-MTT] (Merck, Darmstadt, Germany) to investigate the cytotoxicity of the CDPs against SH-SY5Y (human neuroblastoma) and HEK293 (human embryonic kidney) cells. Both cell lines were purchased from the Leibniz Institute DSMZ-German collection of microorganisms and cell cultures GmbH. HEK293 cells were cultivated in Dulbecco’s modified Eagle’s medium high glucose (Merck, Darmstadt, Germany) containing 2% antibiotic solution of 10 000 units Penicillin, 10 mg streptomycin/ml (Merck, Darmstadt, Germany), and 10% fetal bovine serum (Merck, Darmstadt, Germany) at 37 °C with 5% CO_2_. SH-SY5Y cells were also cultivated in Dulbecco’s modified Eagle’s medium high glucose containing 1% antibiotic solution of 10 000 unit Penicillin,10 mg streptomycin/ml, and 20% fetal bovine serum (Sigma-Aldrich) at 37 °C with 5% CO_2_.

According to the manufacturer’s instructions, cell viability was measured using the Cell Proliferation Kit I (Roche, Basel, Switzerland). The absorbance of the formazan product was determined by measuring the absorption at 570 nm and subtracted from the absorbance at 660 nm.

The SH-SY5Y cells with a density of 10 000 cells (for HEK293 5000 cells) per well (total volume of 100 μL) were added to each well of a 96-well tissue culture plate (VWR North American) and incubated overnight at 37 °C with 5% CO_2_. Following seeding, the cells were subjected to treatment by incubation with varying peptide concentrations ranging from 0 to 100 μM. As a negative control, Triton X-100 was added to five wells, resulting in a final concentration of 0.1%. Subsequently, the plates were placed in a humidified 5% CO_2_ incubator at 37 °C overnight. The next day, 10 μL of MTT labeling reagent from the cell viability kit (Roche, Basel, Switzerland) was added to each well. After 4 h of incubation with the MTT labeling reagent, 100 μL of solubilization buffer was added to each well. The plates were then incubated overnight to ensure complete solubilization of the formazan crystals. Finally, the absorbance was measured at 570 and 660 nm using a CLARIO star plate reader (BMG labtech, Ortenberg, Germany), and the cell viability was calculated using eq 2:
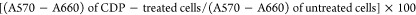
2

A represents the absorbance readings taken from the wells, likely measured using a spectrophotometer or a microplate reader. These absorbance readings are indicative of the metabolic activity of the cells and can be used to assess the cell viability or proliferation. All measurements were performed in triplicate (*n* = 3), and data are presented as mean ± SM.

### Statistical Analysis

For statistical analysis, GraphPad Prism 8.1 was used. For the ThT assay and cell viability assay, two-way ANOVA and Tukey’s test were performed between the groups at different times. For the QIAD assay, two-way ANOVA and Tukey’s test was performed between the groups in fractions 4–6. For the sFIDA assay, a two sample *t* test was used.

## Supporting Information Available

HPLC chromatograms of CDP-1 to -4; HPLC chromatograms of CDP-5 to -8 and CDP-1D; effect of CDP-3, -4, -5, and -7 on Aβ42 aggregation using Thioflavin T assays; dose dependency of CDP-1 and CDP-2 against the Aβ42 aggregation; sFIDA Aβ aggregate control; Biacore SPR kinetic analyses of peptides to Aβ_42_; MTT assay of CDPs on SH-SY5Y cells; MTT assay of CDPs on HEK293 cells; CD spectra of CDPs; negatively charged residues in the Aβ structure and surface; Secondary structure content of CDPs, based on CD-experiments (PDF)
