# Supplementary material for: A Snake Venom Peptide and Its Derivatives Prevent Aβ42 Aggregation and Eliminate Toxic Aβ42 Aggregates In Vitro
Source: ACS Chem Neurosci. 2024 Jul 3;15(14):2600–11. doi: 10.1021/acschemneuro.4c00089 (PMC11258689; doi:10.1021/acschemneuro.4c00089)
Supplement: Supplementary file 1 — cn4c00089_si_001.pdf [file cn4c00089_si_001.pdf]

# Supplementary Information

## A snake venom peptide and its derivatives prevent Aβ$_{42}$ aggregation and eliminate toxic Aβ$_{42}$ aggregates *in vitro*

Luana Cristina Camargo[1,2], Ian Gering[1], Mohammadamin Mastalipour[2], Victoria Kraemer-Schulien[1], Tuyen Bujnicki[1], Dieter Willbold[1,2], Mônika A. Coronado[1,2*], Raphael J. Eberle[1,2*]

[1]Institute of Biological Information Processing (IBI-7: Structural Biochemistry), Forschungszentrum Jülich, 52428 Jülich, Germany.

[2]Heinrich Heine University Düsseldorf. Faculty of Mathematics and Natural Sciences, Institute of Physical Biology, 40225 Düsseldorf, Germany.

Correspondence: monikacoronado@gmail.com (M.A.C.); r.eberle@fz-juelich.de (R.J.E.)

### Table of contents

Figure S1. HPLC chromatograms of CDP-1 to -4.

Figure S2. HPLC chromatograms of CDP-5 to -8 and CDP-1D.

Figure S3. Effect of CDP-3, -4, -5 and -7 on Aβ$_{42}$ aggregation using ThioflavinT assays.

Figure S4. Dose dependency of CDP-1 and CDP-2 against the Aβ$_{42}$ aggregation.

Figure S5. sFIDA Aβ aggregate control.

Figure S6. Biacore SPR kinetic analyses of peptides to Aβ$_{42}$.

Figure S7 MTT assay of CDPs on SH-SY5Y cells.

Figure S8. MTT assay of CDPs on HEK293 cells.

Figure S9. CD spectra of CDPs.

Figure S10. Negatively charged residues in the Aβ structure and surface.

Table S1. Secondary structure content of CDPs, based on CD-experiments

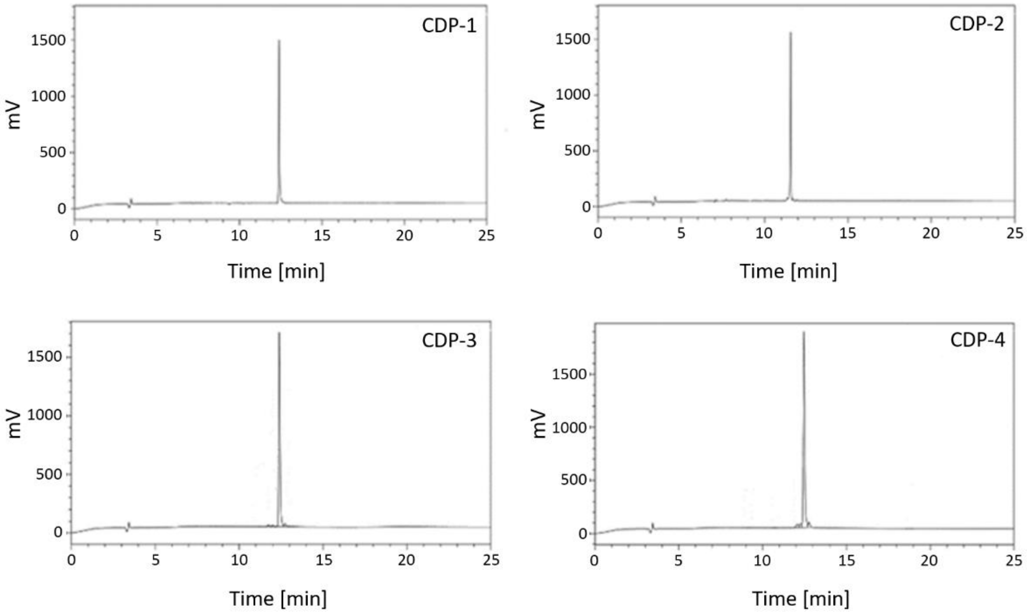


**Figure S1. HPLC chromatograms of CDP-1 to CDP-4.**

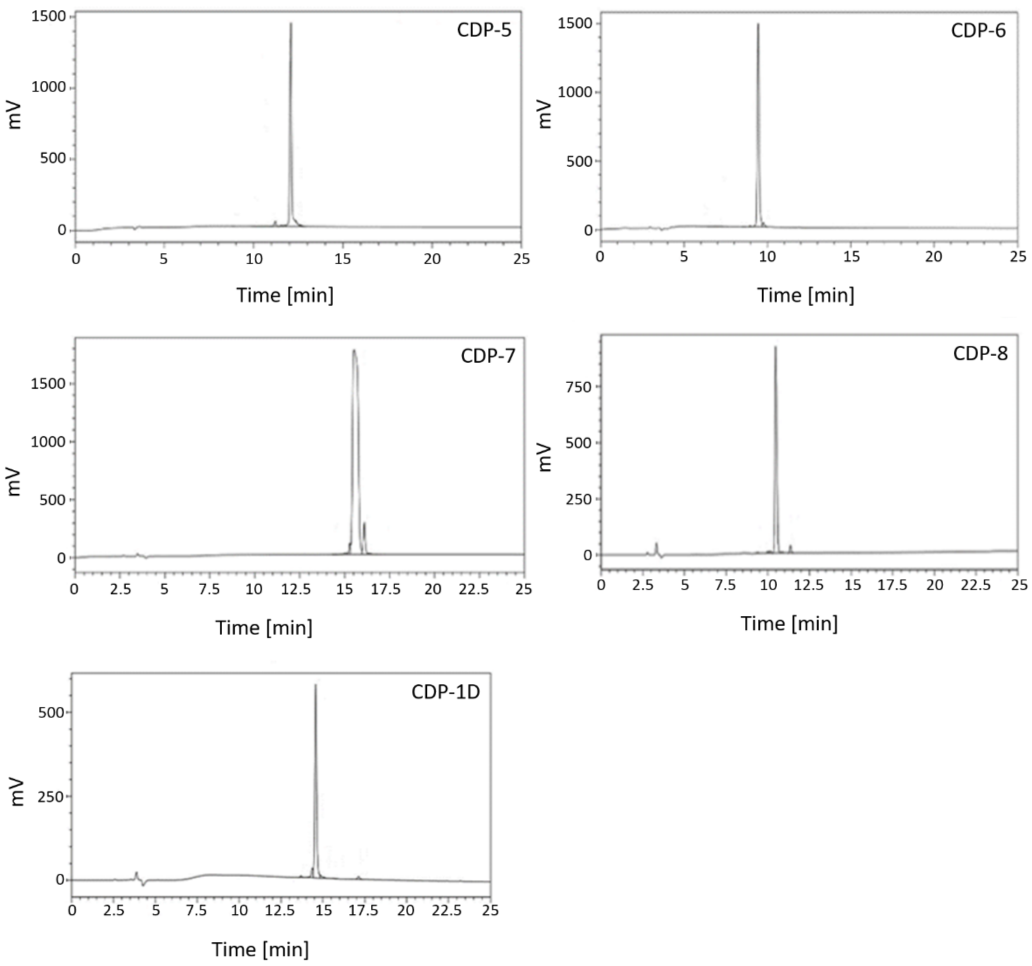


**Figure S2. HPLC chromatograms of CDP-5 to CDP-8 and CDP-1D.**

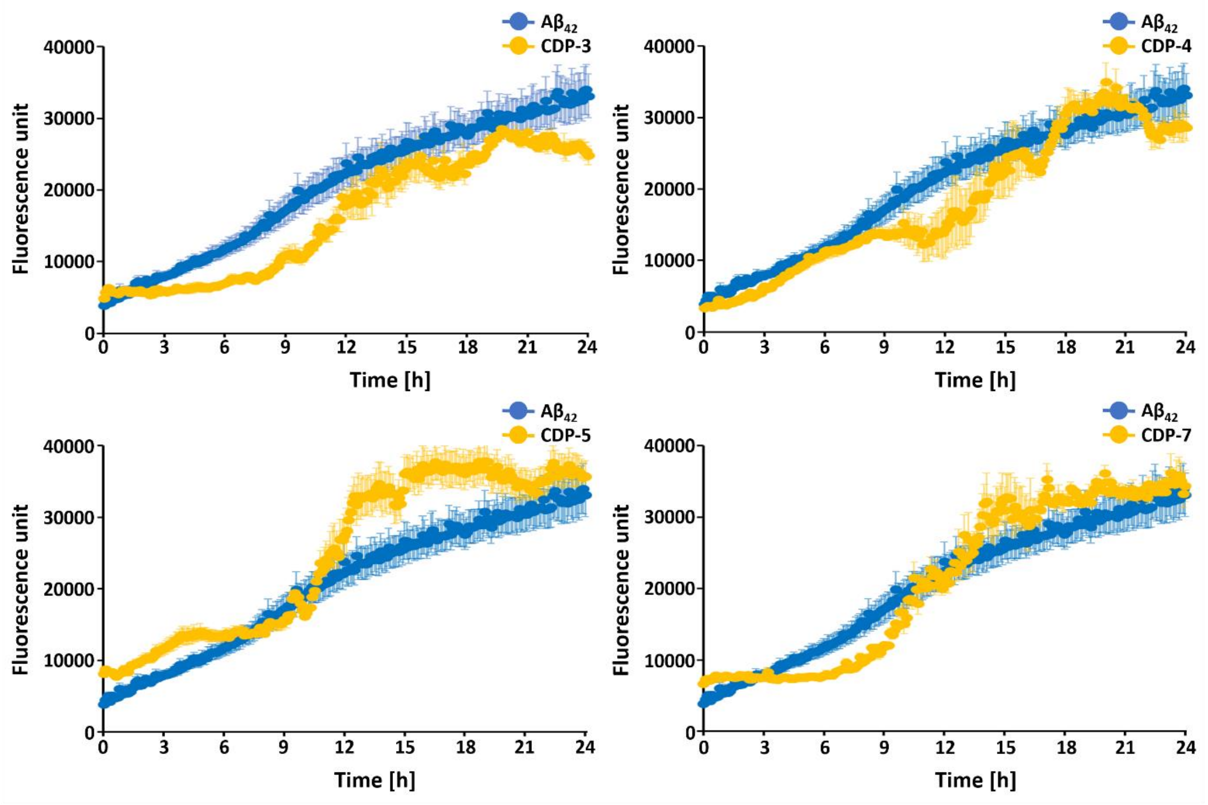


**Figure S3. Effect of CDP-3, -4, -5 and -7 on Aβ42 aggregation using ThioflavinT assays.** The ThT fluorescence signal with only Aβ42 is shown in blue. In orange, the action of CDP-3, -4, -5 and -7 in the signal of ThioflavinT. **A:** Effect of CDP-3 against Aβ42 aggregation. **B:** Effect of CDP-4 against Aβ42 aggregation. **C:** Effect of CDP-5 against Aβ42 aggregation and **D:** Effect of CDP-7 against Aβ42 aggregation. Data shown are the mean ± SEM from three independent measurements (n = 3).

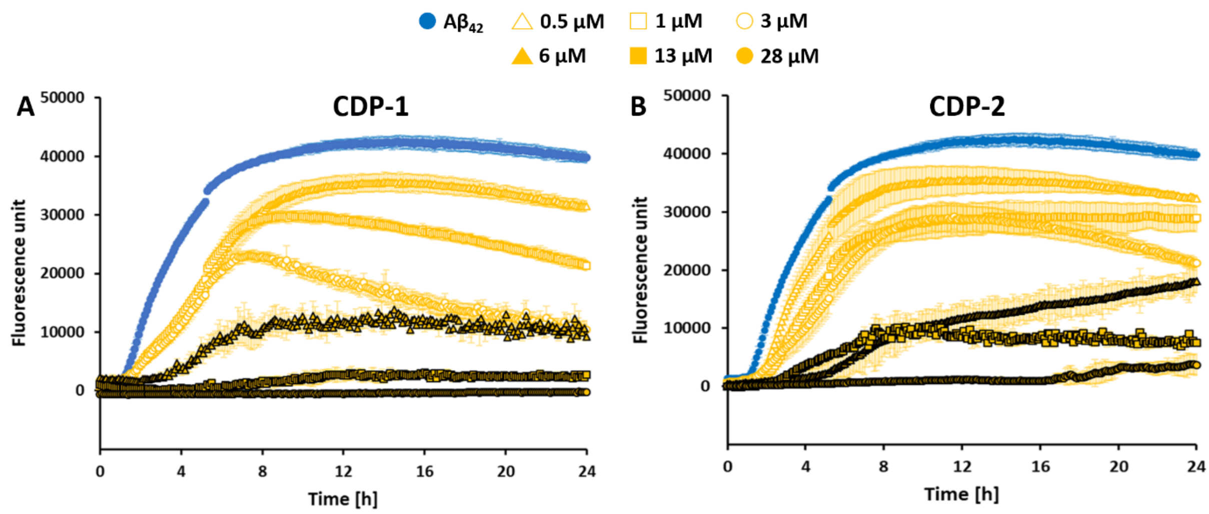


**Figure S4. Dose dependency of CDP-1 and CDP-2 against the Aβ42 aggregation.** The ThT fluorescence signal with only Aβ42 is shown in blue. In orange, the action of CDP-1 and -2 at different concentrations in the signal of ThioflavinT. Effect CDP-1 (**A**) and CDP-2 (**B**) doses (0.5, 1, 3, 6, 13 and 28 µM) on Aβ42 aggregation. Data shown are the mean ± SEM from three independent measurements (n = 3).

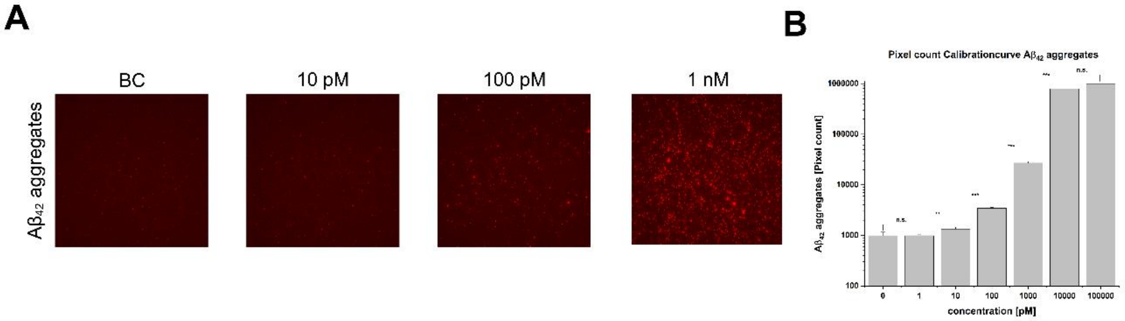


**Figure S5. sFIDA with Aβ aggregates in different concentrations. A:** TIRM images and **B:** Pixel count of the Aβ aggregate standard curve. Data shown are the mean ± SEM from three independent measurements (n = 3). Asterisks mean that the data differ from the control significantly at *: $p<0.05$, **: $p<0.01$ and ***: $p<0.001$ levels according to analyses by a two sample t-test.

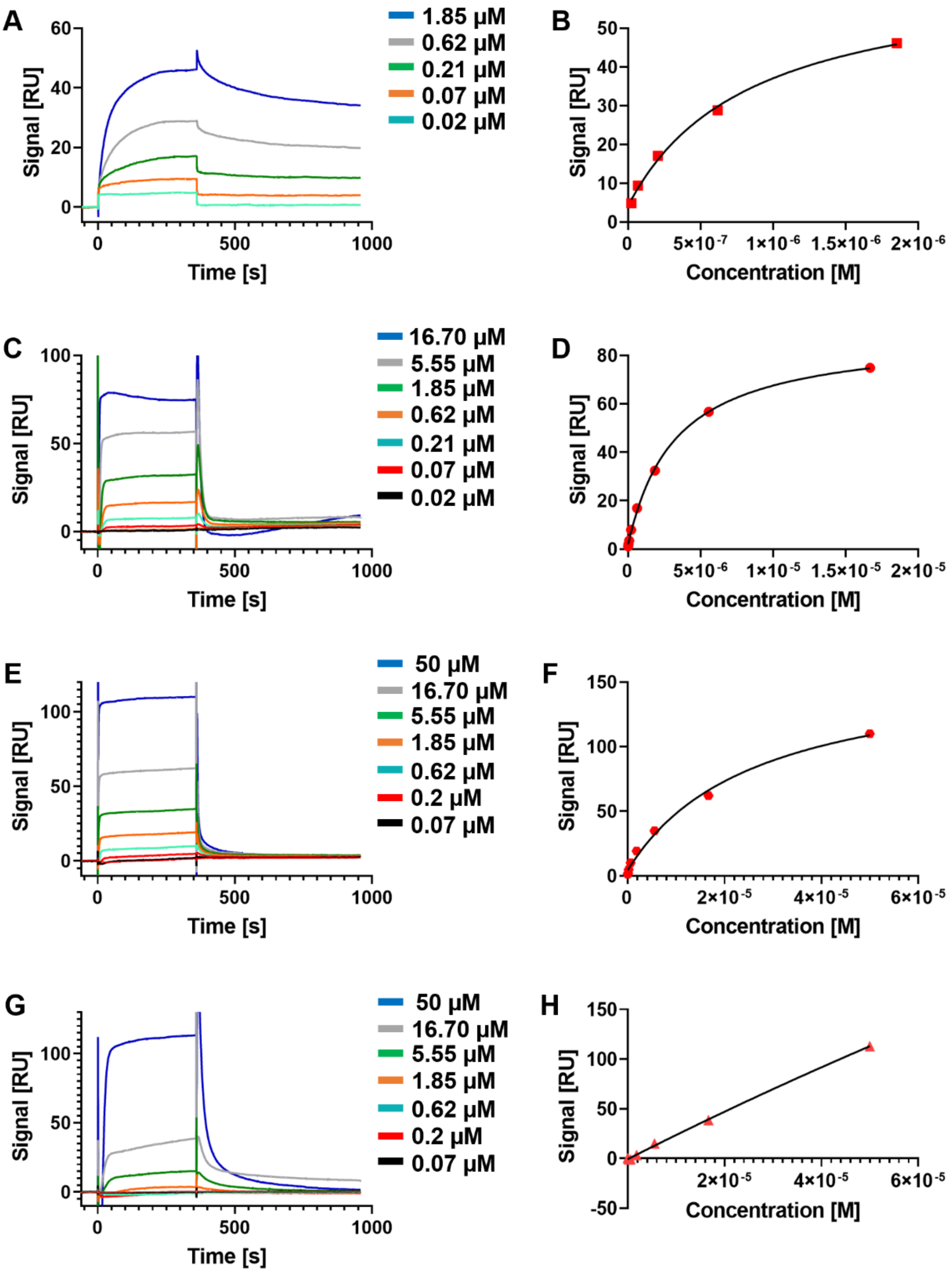


**Figure S6. Biacore SPR kinetic analyses of peptides to $A\beta_{42}$.** The sensorgram and saturation curve of the titration are shown. Sensorgrams were obtained by using a different concentration of peptides (Coloured sensorgrams represent different concentrations in µM). Binding curves were fitted to a steady-state affinity model to get $K_D$ values. CDP-1 (**A**, **B**), CDP-2 (**C**, **D**), CDP-6 (**E**, **F**) and CDP-8 (**G**, **H**).

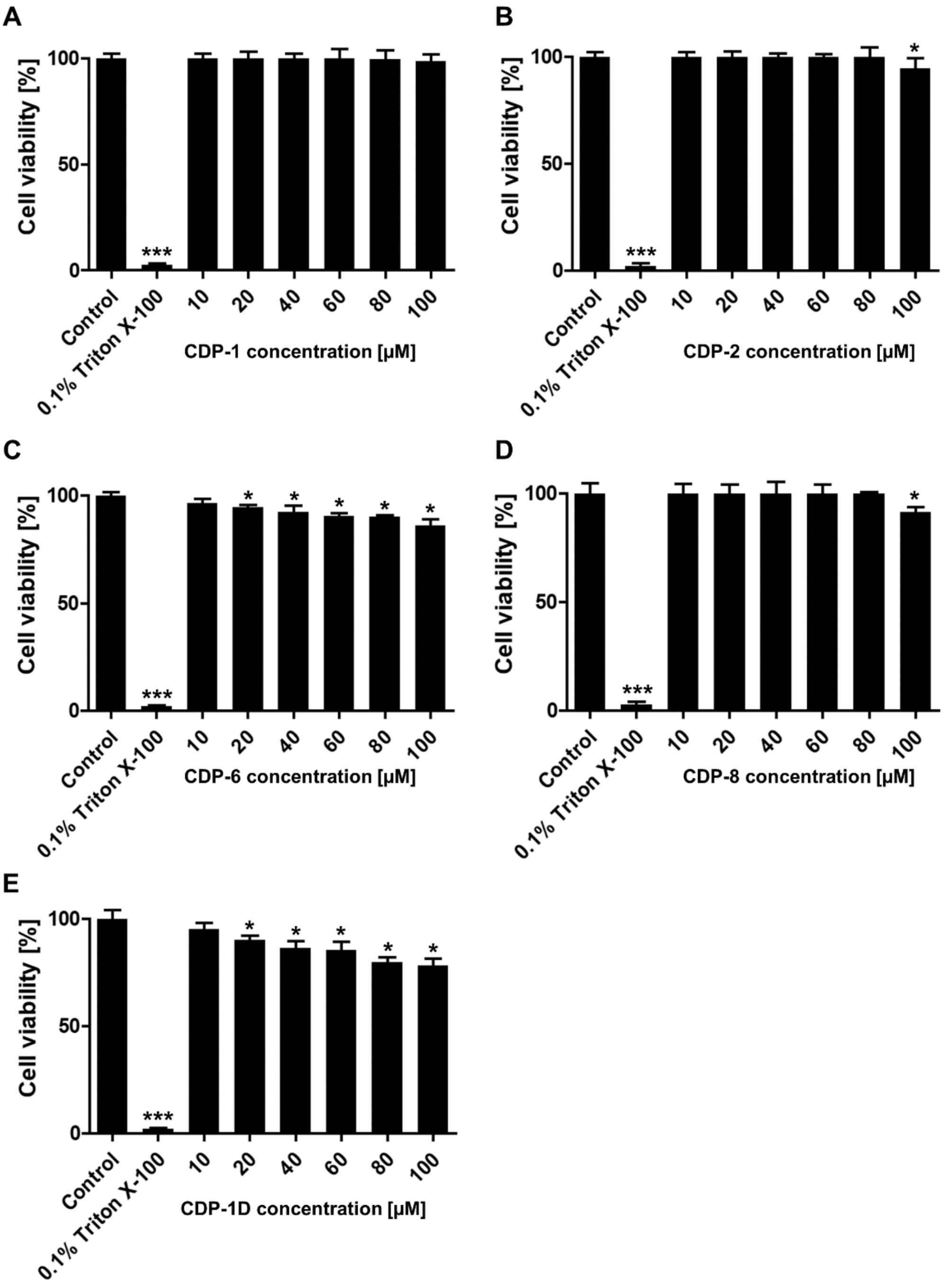


**Figure S7. MTT assay of CDPs on SH-SY5Y cells.** MTT assay evaluated the cytotoxicity of four L-peptides and one D-peptide, each at a concentration range between 0 to 100 µM. The control shows the cell viability without peptide, and 0.1% Triton x-100 was used as a negative control. **A:** CDP-1, **B:** CDP-2, **C:** CDP-6, **D:** CDP-8 and **E:** CDP-1D. Data shown are the means ± SD from three independent measurements (n = 3). Asterisks mean that the data differ from the control significantly at *: p<0.05 and ***: p<0.001 levels according to analyses by two-way ANOVA.

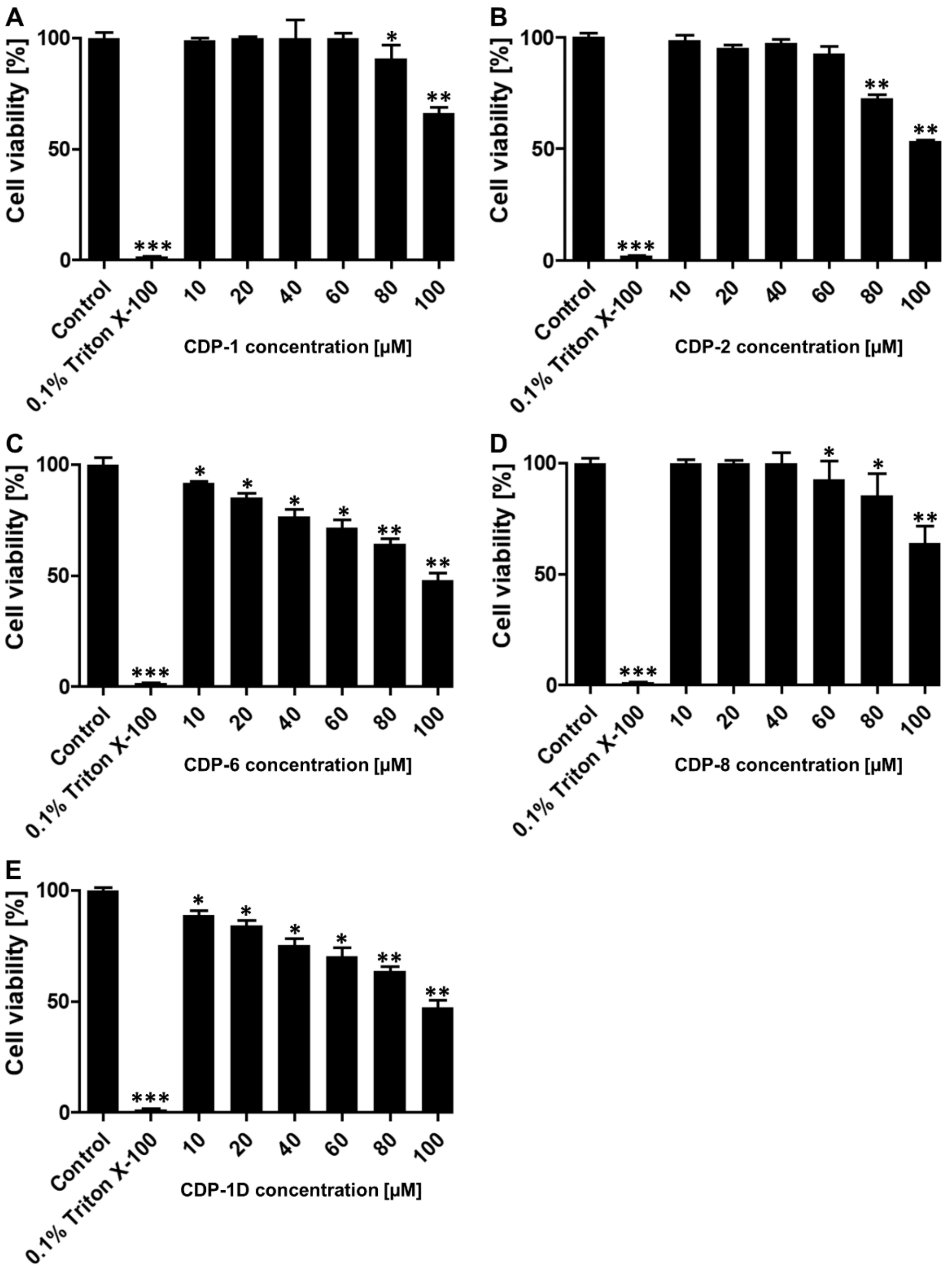


**Figure S8. MTT assay of CDPs on HEK293 cells.** MTT assay evaluated the cytotoxicity of four L-peptides and one D-peptide, each at a concentration range between 0 to 100 μM. The control shows the cell viability without peptide, and 0.1% Triton x-100 was used as a negative control. **A:** CDP-1, **B:** CDP-2, **C:** CDP-6, **D:** CDP-8 and **E:** CDP-1D. Data shown are the means ± SD from three independent measurements (n = 3). Asterisks mean that the data differ from the control significantly at *: $p<0.05$, **: $p<0.01$ and ***: $p<0.001$ levels according to analyses by two-way ANOVA.

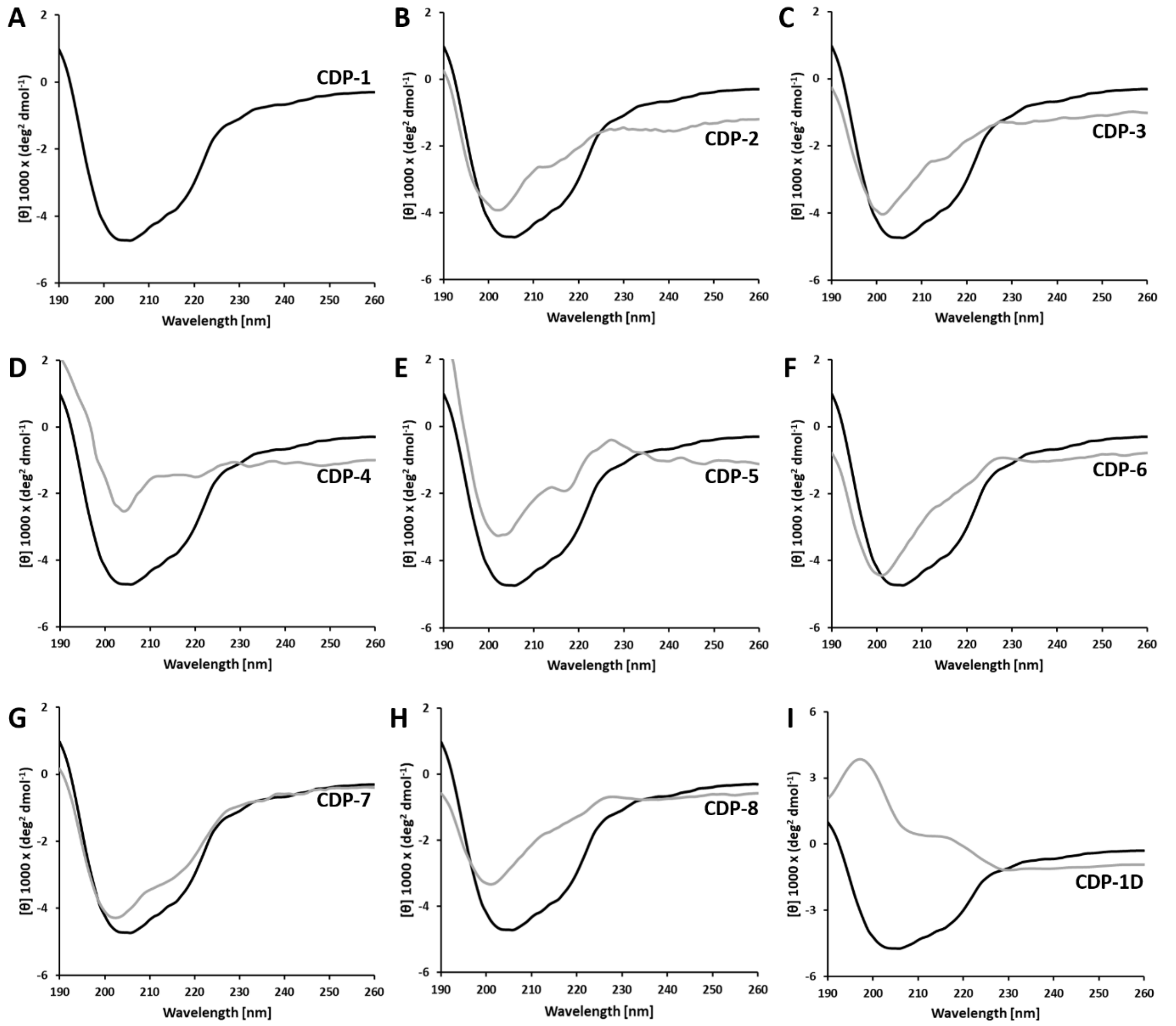


**Figure S9. CD-spectra of CDPs.** The CD spectra of the peptides are shown in reference to the spectrum of CDP-1. All peptides were solved in $ddH_2O$, and the peptide concentration was 30 µM. **A:** CDP-1, **B:** CDP-2, **C:** CDP-3, **D:** CDP-4, E: CDP-5, **F:** CDP-6, **G:** CDP-7, **H:** CDP-8 and **I:** CDP-1D.

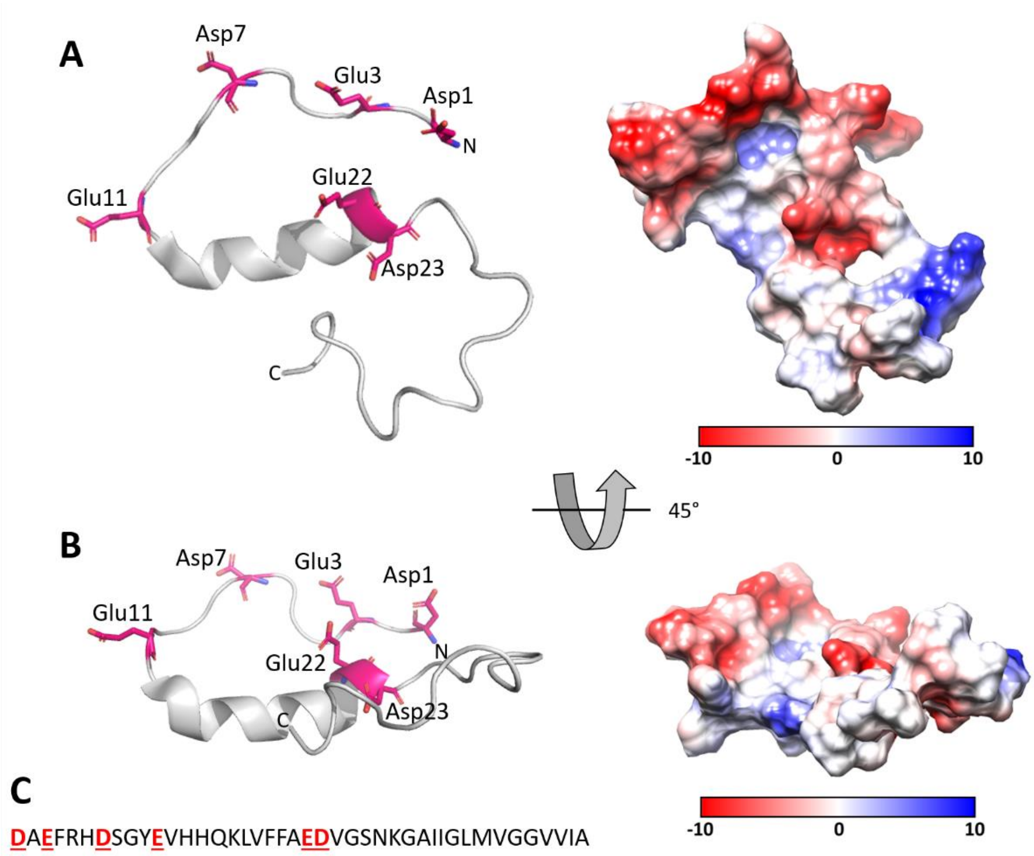


**Figure S10. Negatively charged residues in the Aβ structure and surface**. Ribbon view and coloumbic surface representation of the Aβ monomer structure (PDB: 2LFM). Residues with negative charges are highlighted as sticks. **A:** Ribbon and surface view of the Aβ monomer and **B:** bent forward 45°. **C:** Sequence of Aβ42, the negatively charged residues are highlighted.

**Table S1. Secondary structure content of CDPs, based on CD-experiments[1].**

| | Secondary structure | | |
|---|---|---|---|
| Peptides | α-helix [%] | β-strand [%] | Others [%] |
| CDP-1 | 25 | - | 75 |
| CDP-2 | 3 | - | 97 |
| CDP-3 | 4 | - | 96 |
| CDP-4 | 7 | - | 93 |
| CDP-5 | 4 | - | 96 |
| CDP-6 | 6 | - | 94 |
| CDP-7 | 8 | - | 92 |
| CDP-8 | 6 | - | 94 |

[1]Secondary structure determination was performed using the online tool BeStSel (https://bestsel.elte.hu/index.php).
